# HIF-2α promotes conversion to a stem cell phenotype and induces chemoresistance in breast cancer cells by activating Wnt and Notch pathways

**DOI:** 10.1186/s13046-018-0925-x

**Published:** 2018-10-19

**Authors:** Yuanyuan Yan, Fangxiao Liu, Li Han, Lin Zhao, Jianjun Chen, Olufunmilayo I Olopade, Miao He, Minjie Wei

**Affiliations:** 1 Department of Pharmacology, School of Pharmacy, China Medical University, No.77 Puhe Road, Shenyang North New Area, Shenyang City, 110122 Liaoning China; 2 Liaoning Key Laboratory of molecular targeted anti-tumor drug development and evaluation, Shenyang North Area, Shenyang City, 110122 Liaoning China; 3 Department of Systems Biology, City of hope, Los Angeles, CA USA; 4 Center for Clinical Cancer Genetics, Department of Medicine, The University of Chicago, Chicago, IL USA

**Keywords:** Hypoxia-inducible factor-2α, Stemness phenotype, Wnt pathway, Notch pathway, Drug resistance

## Abstract

**Background:**

Hypoxic tumor microenvironment and maintenance of stemness contribute to drug resistance in breast cancer. However, whether Hypoxia-inducible factor-2α (HIF-2α) in hypoxic tumor microenvironment mediates conversion to a stem cell phenotype and chemoresistance of breast tumors has not been elucidated.

**Methods:**

The mRNA and protein expressions of HIF-1α, HIF-2α, Wnt and Notch pathway were determined using qRT-PCR and western blot. Cell viability and renew ability were assessed by MTT, Flow cytometric analysis and soft agar colony formation.

**Results:**

In our study, acute hypoxia (6–12 h) briefly increased HIF-1α expression, while chronic hypoxia (48 h) continuously enhanced HIF-2α expression and induced the resistance of breast cancer cells to Paclitaxel (PTX). Furthermore, HIF-2α overexpression induced a stem cell phenotype, the resistance to PTX and enhanced protein expression of stem cell markers, c-Myc, OCT4 and Nanog. Most importantly, Wnt and Notch signaling, but not including Shh, pathways were both activated by HIF-2α overexpression. Dickkopf-1 (DKK-1), a Wnt pathway inhibitor, and L685,458, an inhibitor of the Notch pathway, reversed the resistance to PTX and stem phenotype conversion induced by HIF-2α overexpression. In addition, HIF-2α overexpression enhanced tumorigenicity and resistance of xenograft tumors to PTX, increased activation of the Wnt and Notch pathways and induced a stem cell phenotype in vivo.

**Conclusion:**

In conclusion, HIF-2α promoted stem phenotype conversion and induced resistance to PTX by activating Wnt and Notch pathways.

**Electronic supplementary material:**

The online version of this article (10.1186/s13046-018-0925-x) contains supplementary material, which is available to authorized users.

## Background

Breast cancer is one of the most common cancers in women worldwide. The incidence rate of breast cancer has been reported to be 30%, ranking first, and the death rate was stated as 14%, ranking second just behind lung cancer in 2017 [1]. Breast cancer is said to be the leading cause of cancer deaths in China [2]. Although paclitaxel (PTX) is one of the first-line chemotherapeutic drugs used to treat breast cancer, chemoresistance to PTX is a major challenge in achieving successful chemotherapy results [3].

Breast cancer stem cells (BCSCs) in the tumor microenvironment are considered the seeds of cancer formation and play pivotal roles in drug resistance and tumor relapse [4, 5]. Wnt and Notch pathways are master developmental pathways that perform central roles in the development of cancer stem cells and stemness maintenance [6]. The Wnt pathway, which is transduced by LRP 5/6 and Frizzled receptor complexes, results in inhibition of β-catenin destruction, and β-catenin migration to the nucleus activates target genes, such as c-Myc and survivin [6, 7]. Activation of the Notch signaling pathway after two proteolytic events leads to the release of Notch intracellular domain (Notch^NIDC^), an intracellular active form of the receptors, and promotes the expression of target genes, including c-Myc and the Hey family [8, 9]. Proto-oncogene c-Myc, which is a common target gene of Wnt and Notch pathways, is an important transcription factor to induce stem cell-like traits and preserve cancer stemness [10]. Furthermore, c-Myc drives tumor development and drug resistance in cancer cells, including breast cancer [11, 12].

A hypoxic tumor microenvironment regulates the stemness phenotype in BCSCs. One major mechanism of stemness maintenance induced by hypoxia is activation of hypoxia-inducible factors (HIFs) [13, 14]. HIFs are basic helix-loop-helix-Per-ARNT-Sim (bHLH-PAS)-containing transcription factors and consist of a heterodimer of an O_2_-regulated α subunit (HIF-1α, HIF-2α and HIF-3α) and a constitutively expressed subunit [15]. Variations in hypoxic tumor microenvironments, for example acute hypoxia and chronic hypoxia, can contribute to different stabilities of HIF-1α and HIF-2α [16]. Though HIF-1α and HIF-2α are highly homologous, they regulate distinct target genes and usually play different roles in proliferation, invasion and chemotherapeutic resistance [17, 18]. HIF-1α mainly mediates angiogenesis, metabolic reprogramming, invasion and metastasis and epithelial-mesenchymal transition to promote cancer tumorigenesis [19]. HIF-2α is an important factor in promoting a cancer stem cell state and shows specificity and selectivity to cancer stem cells [20–23]. It was recently reported that HIF-2α can regulate self-renewal ability and maintain immature phenotypes in BCSCs [24]. Whether HIF-2α can mediate breast cancer cell conversion to BCSCs to induce PTX resistance and related mechanisms are still unclear.

In this study, we found that chronic hypoxia enhanced the resistance of breast cancer cells to PTX and induced high expression of HIF-2α, but not HIF-1α. Furthermore, we demonstrated that HIF-2α promoted the stem cell properties of breast cancer cells with increased expression of c-Myc, OCT4 and Nanog and induced resistance to PTX by activating Wnt and Notch signaling pathways.

## Methods

### Cell lines and chemical reagents

MCF7 and MDA-MB-231 human breast carcinoma cell lines were obtained from ATCC (Manassas, VA, USA). MCF7 and MDA-MB-231 cells were maintained in high-glucose (4.5 mg/ml) DMEM (HyClone) or L15 (Gibco) medium with 10% (*v*/v) FBS (HyClone). MCF7 mammosphere (MCF7 MS) cell lines were cultured in DMEM-F12 medium supplemented with 2% B27 (Gibco, Thermo Fisher Scientific), b-FGF 10 μg/L (Promega), EGF 20 μg/L (Promega). For hypoxia treatment, MCF7 cells were cultured under continuous reduced oxygen conditions (1%) using a HERACELL 150i CO2 Incubator (Thermo Fisher Scientific Inc). Dickkopf-1 (DKK-1) was purchased from StemRD. Paclitaxel, L685458 and PEG-35 castor oil were obtained from Sigma.

### Generation of stable HIF-2α-overexpressing cells

Lentiviral vectors (GV358) were purchased from Shanghai Genechem Co., Ltd. HIF-2α (NM_001430) cDNA was cloned into a Ubi-MCS-3FLAG-SV40-EGFP-IRES-puromycin vector (Genechem). MCF7 and MDA-MB-231 cells were seeded into 6-well plates at a density of 1 × 10^5^ cells per well. The next day, the culture medium was removed and the lentiviral vectors and polybrene were mixed with medium at MOI = 10 for NC-cDNA and MOI = 20 for HIF-2α-cDNA. After transfection for 12 h, fresh culture medium was added and culturing continued for 48 h. Puromycin (1μg/ml) was then added into the medium to select stably transfected cells. This process was repeated 2 to 3 times until all cells expressed green fluorescent protein (GFP).

### Generation of stable HIF-2α-silencing cells

Lentiviral vectors (GV115) were purchased from Shanghai Genechem Co., Ltd. HIF-2α-RNAi (NM_001430) sequence is GTTCTGGTGACTCTTGGTC (Genechem). MCF7 MS cells were seeded into 6-well ultra-low adhesion plates (Corning) at a density of 2 × 10^5^ cells per well. The next day, lentiviral vectors and polybrene were mixed with medium (sh-Ctrl, MOI = 20; sh-HIF-2α, MOI = 25). After transfection for 12 h, fresh culture medium was added, and culturing continued for 48 h. Puromycin (2 μg/ml) was added to select stably transfected cells. This process was repeated 2 to 3 times until all cells expressed green fluorescent protein (GFP).

### Quantitative real-time PCR

Total RNA was extracted from MCF7 cells using Trizol (Invirogen, USA). cDNA was compounded using the PrimeScript™ RT reagent Kit with gDNA Eraser (Takara, Japan). Real-time PCR was performed to measure *HIF-1A* (HIF-1α) and *EPAS1* (HIF-2α) expression using SYBR® Green Realtime PCR Msater Mix (TOYOBO, Japan). Fold change of *HIF1A* and *EPAS1* was calculated using the 2^-ΔΔCt^ method. Primers used in this study were below: *EPAS1* forward: CTACGCCACCCAGTACCAGG, *EPAS1* reverse: GACACCTTGTGGGCTGACG, *HIF-1A* forward: ACCATGCCCCAGATTCAGG, *HIF-1A* reverse: AGTGCTTCCATCGGAAGGACT.

### Western blot

Cells were washed with cold PBS and lysed in RIPA buffer containing 1% proteinase inhibitor cocktail solution and 1% phosphatase inhibitor cocktail solution (Sigma-Aldrich). Total protein extracts of 10–30 μg were separated on 8–15% SDS-PAGE gels. After electrophoresis, the proteins were transferred to a polyvinylidene fluoride (PVDF) membrane (Millipore, Bedford, MA). The primary antibodies were HIF-1α (1: 500, CST, #3716), HIF-2α (1: 500, CST, #7096), c-Myc (1: 1000, CST, #5605), Hey2 (1: 1000, Abcam, ab167280), β-catenin (1: 1000, Proteintech, 51067–2-AP), p-β-catenin (1: 1000, CST, #9561), Axin2 (1: 1000, CST, #2151), Survivin (1: 1000, CST, #2808), Notch^NICD^ (1: 1000, CST, #4147), OCT4 (1: 1000, CST, #2750), and Nanog (1: 1000, CST, #4903).

### Cell viability assay

NC-cDNA or HIF-2α-overexpressing MCF7 and MDA-MB-231 cells were seeded into 96-well plates (5.0 × 10^3^ cells per well). Cell viability was assessed by MTT (Sigma). To determine the IC_50_ value of PTX, cells were treated with PTX (0–300 nM for MCF7 and 0–30000 nM for MDA-MB-231) under normoxia (20% O_2_) or hypoxia (1% O_2_) for 6–72 h. The absorbance was monitored by an Anthos 2010 microplate reader (Anthos Labtec Instruments) at 570 nm.

### Soft agar colony formation assay

The soft agar colony formation assay was following previous study [25], 6-well plates were coated with a bottom layer of 1.2% SeaPlaque low melting temperature agarose (Lonza Rockland, ME USA) in phenol red-free medium supplemented with 20% FBS. Ten thousand cells were mixed in 0.6% agarose and the same medium and applied as the top agarose layer. The top agarose layer was overlaid with 600 μl medium. The plates were incubated at 37 °C in 5% CO_2_ for 3 weeks until colonies formed. The colonies were stained with 100 μl MTT (5 mg/ml) to each well and incubated for 30 min at 37 °C. 20 days later, colonies larger than 0.2 mm in diameter were counted. Colonies were counted using the analysis software Quantity One (BioRad, Hercules, California, USA).

### Mammosphere formation assay

After treated with PTX for 48 h, MCF7 MS cells (2000 cells/ml) were culture in ultra-low adhesion plates (Corning) in DMEM-F12 (GIBCO), containing 2% B27 (Gibco, Thermo Fisher Scientific), b-FGF 10 μg/L (Promega), EGF 20 μg/L (Promega). After culturing for 14 days, mammosphere with diameter > 150 μm were counted. Six replicate wells were included in each analysis and at least three independent experiments were conducted.

### TUNEL

The effect of PTX treatment in vivo was further evaluated by deoxynucleotidyl transferase-mediated dUTP nick end labeling (TUNEL) assay following the manufacturer’s protocol (Key GEN BioTECH). Tumor tissues obtained at the end of experiments were fixed in formalin, dried in ethanol, cut into 10-μm thick slices, and prepared for the TUNEL assay. TUNEL-positive cells were identified by red fluorescence of the nuclei, which were counted and compared across treatment groups. Ten independent microscopic fields-of-view were manually quantified and used for statistical comparisons.

### Flow cytometric analysis

For apoptosis analysis, MCF7 and MDA-MB-231 cells were resuspended in cold 300–400 μl annexin V binding buffer at a density of 10^6^ cells/ml. The suspension was incubated in the dark at room temperature for 15 min with a solution of annexin V-FITC (2.5 μg/ml). PI (5 μg/ml) was added 5 min prior to flow cytometric analysis using a FACSCalibur instrument (Becton-Dickinson).

### In vivo xenograft model

HIF-2α-overexpressing MCF7 and MDA-MB-231 cells were mixed with Matrigel (BD Biosciences) at a 1: 1 ratio. 3 × 10^6^ MCF7 cells and 1 × 10^6^ MDA-MB-231 cells were unilaterally injected subcutaneously into the mammary fat pad of 3–4 weeks-old BALB/c (nu/nu) mice. After the tumors reached 125 mm^3^ in size, the mice were randomized into six groups: blank + DMSO, blank + PTX, NC-cDNA + DMSO, NC-cDNA + PTX, HIF-2α-cDNA + DMSO, and HIF-2α-cDNA + PTX. Mice were injected i.p. with PTX (5 mg/kg) and DMSO as a control every other day. Tumor diameters were measured with digital calipers before each treatment. PTX was solubilized in DMSO and PEG-35 castor oil (1: 24). Mice were sacrificed after 20 days of the start of treatment. Before being sacrificed, mice were anesthetized with chloral hydrate then photographed using Living Image software (Perkin-Elmer). All mice were bred in pathogen-free conditions at the Animal Center of China Medical University. All animal studies were performed in accordance with the National Institute of Health Guide for the Care and Use of Laboratory Animals.

### TCGA Chort and GEO data processing

TCGA expression data (245 cases included) were downloaded from TCGA official website (http://cancergenome.nih.gov/), and the GSE47533 (6 cases included) and GSE58383 were (6 cases included) were acquired from GEO website (https://www.ncbi.nlm.nih.gov/geo/). In TCGA, total breast cancer patients were 1168 cases, 245 cases breast cancer patients were selected from TCGA with CD44^+^CD24^−^ phenotype. The positive criterion of CD44 and CD24 were based on the ROC curve. GSE47533 datasets included microRNA sequencing data and gene expression microarray data generated from MCF7 cells submitted to a hypoxia time course (16 h, 32 h and 48 h at 1% oxygen). GSE58383 datasets included transcriptome analysis of MCF7 MS (MCF7 mammospheres) and parental MCF7 breast cancer cell line.

### Statistical analysis

Statistical analyses were conducted in GraphPad Prism 7. Results are presented as the mean ± standard deviation (SD) for at least three experiments. Student’s *t*-test was used to compare differences between two groups. One-way ANOVA was used to compare differences among three or more groups. A *P* value of < 0.05 was considered statistically significant.

## Results

### Chronic hypoxia enhances breast cancer cells chemoresistance to paclitaxel and induces high expression of HIF-2α but not HIF-1α

**Fig. 1:**
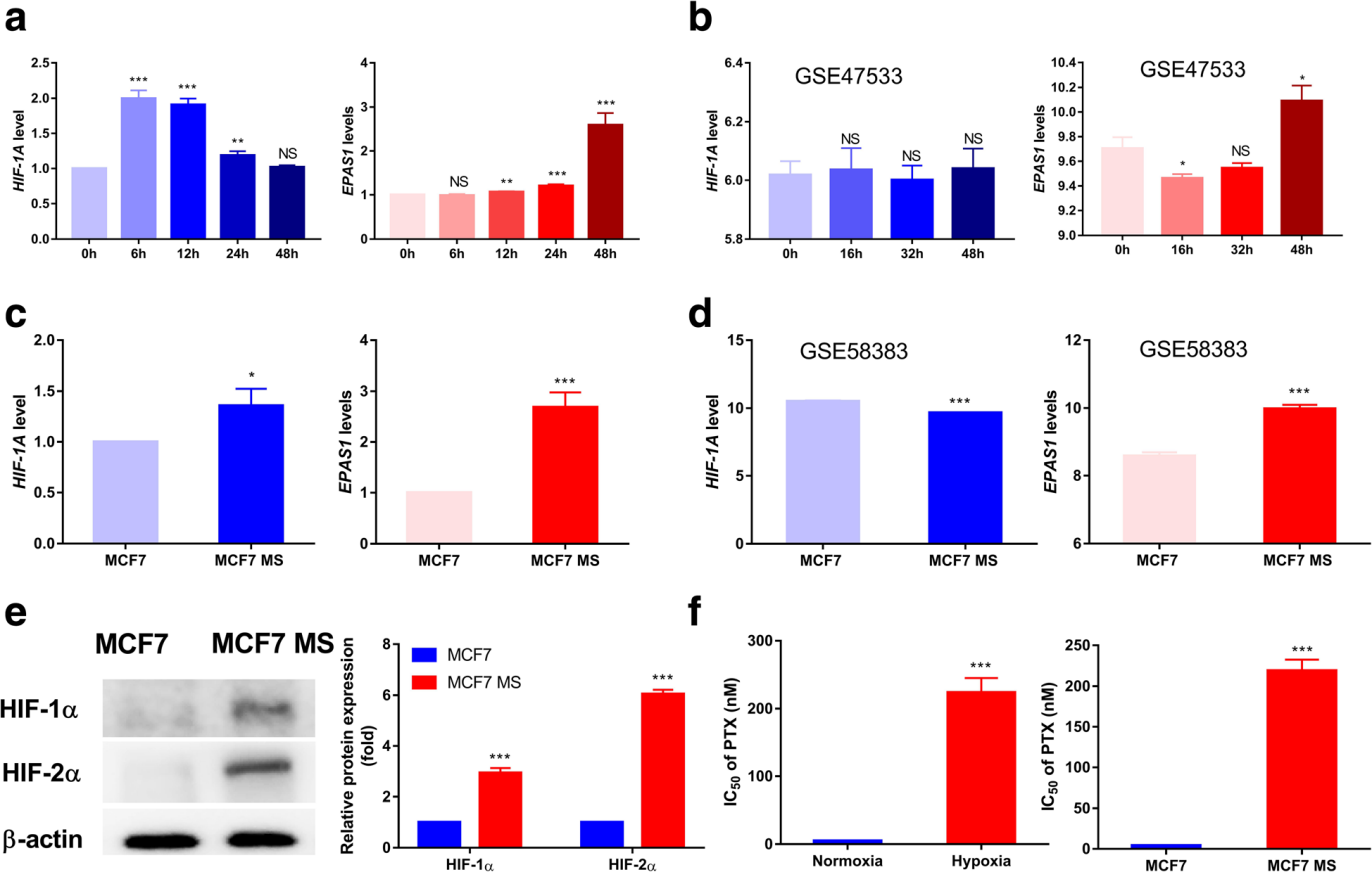
Chronic hypoxia increases breast cancer cell resistance to PTX and HIF-2α expression. **a** mRNA levels of HIF-1α and HIF-2α in MCF7 cells under normoxia or hypoxia for 6–48 h were detected by qRT-PCR. **b** mRNA levels of HIF-1α and HIF-2α in MCF7 cells under normoxia or hypoxia for 16–48 h were analyzed from GSE47533. **c** mRNA levels of HIF-1α and HIF-2α in MCF7 and MCF7 MS cells. **d** mRNA levels of HIF-1α and HIF-2α in MCF7 and MCF7 MS cells from GSE58383. **e** Protein levels of HIF-1α and HIF-2α in MCF7 and MCF7 MS cells. Statistical significance of detected protein expression is also shown. **f** Left panel: Cell viability of MCF7 cells treated with PTX (0–300 nM) under normoxia (20% O_2_) or hypoxia (1% O_2_) for 48 h was detected by MTT assay. Right panel: Comparison of IC_50_ values of MCF7 and MCF7 MS cells treated with different concentrations of PTX for 48 h. Data are the mean ± SD from three independent experiments. ^*^P < 0.05, ^**^P < 0.01, ^***^P < 0.0001 compared with the normoxia group

Hypoxia microenvironment induces HIFs expression [13]. We detected changes of HIF-1α (*HIF-1A*) and HIF-2α (*EPAS1*) mRNA expression in MCF7 cells under 1% O_2_ hypoxic treatment for 6, 12, 24, and 48 h. The kinetics of HIF-1α and HIF-2α accumulation in response to hypoxia varied (Fig. 1a). HIF-1α expression increased under hypoxia for 6 and 12 h, then gradually declined for 24 and 48 h. In contrast, HIF-2α expression continued to increase until 48 h. we also detected HIF-1α and HIF-2α express from GEO data set. In the series of GSE47533, *HIF-1A* has no obvious changes, but *EPAS1* was obviously increased at 48 h (Fig. 1b). These all implied that chronic hypoxia could induce HIF-2α high expression but not HIF-1α.

Although BCSCs exists in hypoxia microenvironment, how HIFs regulated BCSCs phenotype is still unclear. Hence, we detected mRNA and protein levels of HIF-1α and HIF-2α in MCF7 mammospheres (MCF7 MS) cells and found though HIF-1α and HIF-2α increased but the level of HIF-2α was higher (Fig. 1c, e). Similarly, data from GSE58383 exhibited HIF-2α notably enhanced, but HIF-1α slightly declined in MCF7 MS cells (Fig. 1d).

To investigate how hypoxia impacts drug sensitivity in breast cancer cells, we measured MCF7 cells viabilities when treated with different concentrations of PTX under normoxia (20% O_2_) or hypoxia (1% O_2_) for 48 h by MTT. As shown in Fig. 1f left panel, cell viability of MCF7 cell was obviously enhanced under hypoxic condition for 48 h compared with normoxia. Similarly, MCF7 MS cells viability was also obviously enhanced than that of parental MCF7 cells after the treatment of PTX for 48 h (Fig. 1f right panel). Based on these, we inferred that breast cancer cell resistance to PTX induced by chronic hypoxia may be more related to HIF-2α and stem characteristic.

### HIF-2α overexpression increases stem-like properties and c-Myc expression and induces the resistance of breast cancer cells to paclitaxel

**Fig. 2:**
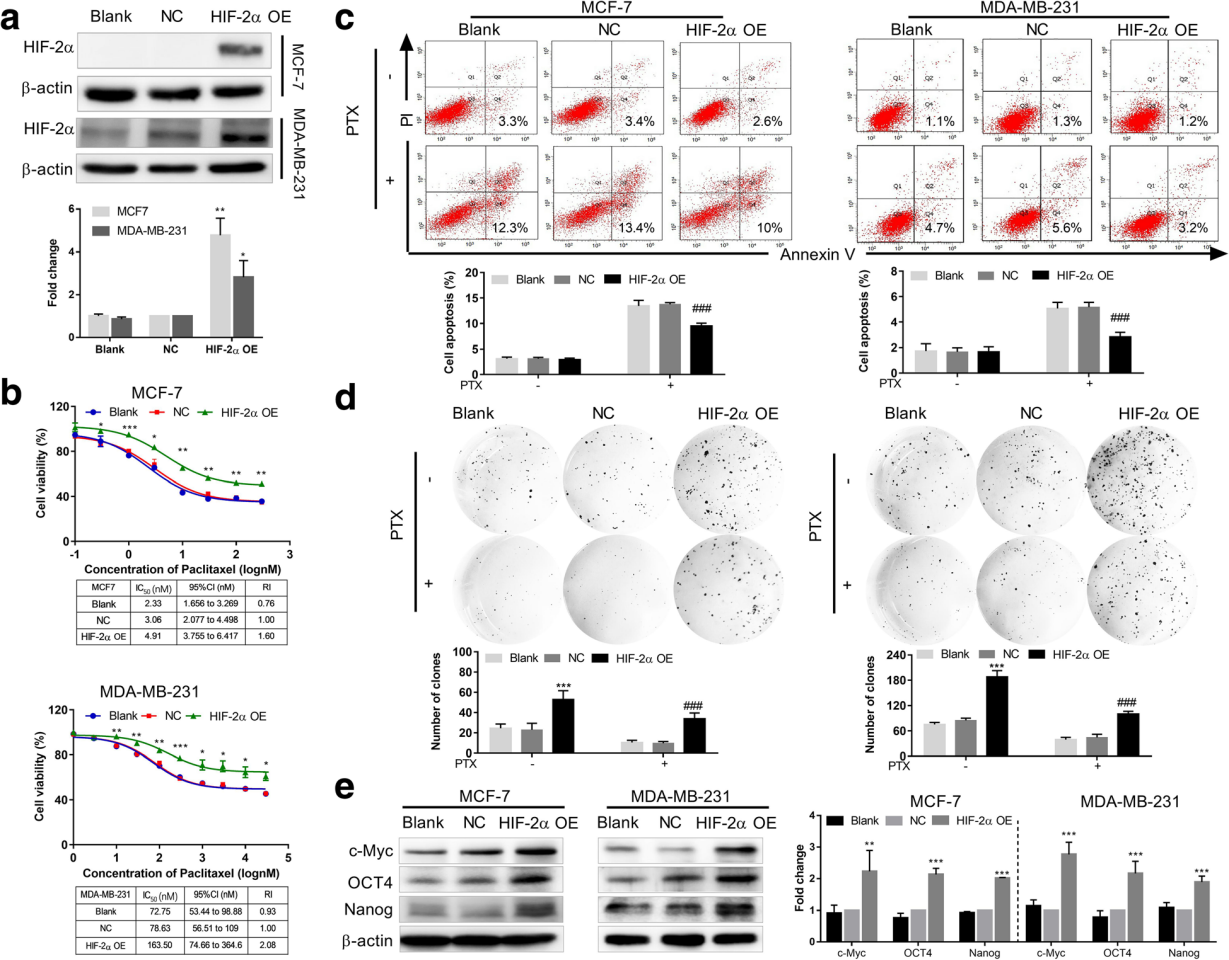
HIF-2α overexpression increases breast cancer cells resistance to PTX, suppresses apoptosis, and up-regulates stem cell marker expression. **a** HIF-2α expression was detected in stably HIF-2α–cDNA transfected MCF7 and MDA-MB-231 cells by western blot. Densitometric analysis of detected protein expression is also shown. **b** Top: Cell viability of stable HIF-2α–cDNA transfected MCF7 and MDA-MB-231 cells 48 h after treatment with PTX (0–300 nM and 0–30000 nM, respectively) was measured using the MTT assay. Bottom: Comparison of IC_50_ values and resistance index (RI). **c** Cell apoptosis was analyzed by flow cytometry after stable HIF-2α–cDNA transfected MCF7 and MDA-MB-231 cells were incubated with PTX (3 nM and 100 nM, respectively) for 48 h. **d** Self-renewal ability was analyzed after stable HIF-2α–cDNA transfected MCF7 and MDA-MB-231 cells were incubated with PTX (3 nM or 100 nM, respectively) for 48 h. **e** Left: Expression of c-Myc, OCT4 and Nanog in HIF-2α–overexpressing MCF7 and MDA-MB-231 cells was detected by western blot. Right: Densitometric analysis of protein expression. Data are the mean ± SD of three independent experiments performed in triplicate. ^*^P < 0.05, ^**^P < 0.01, ^***^P < 0.0001 compared with the NC-cDNA group. ^###^*P* < 0.0001 compared with the NC-cDNA + PTX group

Previous results suggested that the resistance of breast cancer cells to PTX induced by chronic hypoxia may be related to HIF-2α expression. To further confirm this, we first transfected MCF7 and MDA-MB-231 cells with HIF-2α-cDNA lentivirus to construct stable HIF-2α-overexpressing cell models. We observed that HIF-2α protein expression was increased in HIF-2α-cDNA (HIF-2α OE)-transfected MCF7 and MDA-MB-231 cells compared with the negative control (NC) group by western blot (Fig. 2a, *P* < 0.01, *P* < 0.05, respectively), showing that breast cancer cells overexpressing HIF-2α were successfully constructed.

To investigate whether HIF-2α overexpression impacts the drug sensitivity of breast cancer cells to PTX, we treated HIF-2α-overexpressing MCF7 and MDA-MB-231 cells with different concentrations of PTX for 48 h and examined changes in cell viability. Compared with the NC group, HIF-2α-cDNA-transfected MCF7 and MDA-MB-231 cells exhibited significantly higher cell viability with increased IC_50_ values and resistance index (RI) (Fig. 2b). In addition, we detected cell apoptosis after PTX treatment (3 nM and 100 nM for MCF7 and MDA-MB-231 cells, respectively) for 48 h by flow cytometry. Apoptosis rates were significantly decreased in HIF-2α-cDNA-transfected MCF7 and MDA-MB-231 cells compared to the NC group (Fig. 2c, *P* < 0.001). These results indicate that HIF-2α overexpression enhanced the drug resistance of MCF7 and MDA-MB-231 cells to PTX.

To further study whether HIF-2α overexpression impacts the stem cell phenotype, we detected the self-renewal ability of MCF7 and MDA-MB-231 cells after PTX treatment for 48 h. Compared with NC group, HIF-2α-cDNA-transfected MCF7 and MDA-MB-231 cells formed more and bigger colonies (Fig. 2d). To study the roles of stem phenotype in the resistance of breast cancer cells to PTX mediated by HIF-2α, we assessed changes in c-Myc, OCT4, Nanog, the stem cell markers, protein expression after overexpressing HIF-2α. Compared with the NC group, c-Myc, OCT4, Nanog expressions were increased in HIF-2α-cDNA-transfected MCF7 and MDA-MB-231 cells (Fig. 2e). We also detected c-Myc, OCT4 and Nanog expressions with or without PTX treatment (3 nM and 100 nM for MCF7 and MDA-MB-231 cells, respectively) for 48 h. As shown in Additional file 1: Figure S1, there is no obvious difference between with PTX and without PTX treatment.

Furthermore, to verify the function of HIF-2α in stem-like characteristics, we constructed stably HIF-2α silencing MCF7 MS cells, and detected the changes of chemoresistance and self-renew ability. The data showed that HIF-2α-silenced MCF7 MS cells had obviously decreased chemoresistance and self-renew ability (Additional file 2: Figure S2a, b). We also found that silencing HIF-2α significantly reduced the protein expressions of c-Myc, OCT4 and Nanog in MCF7 MS cells (Additional file 2: Figure S2c).

All these results suggest that HIF-2α could promote stem-like properties and c-Myc expression and induce the resistance of breast cancer cells to PTX.

### HIF-2α overexpression increases stem-like properties and breast cancer cells resistance by activating the Wnt pathway

**Fig. 3:**
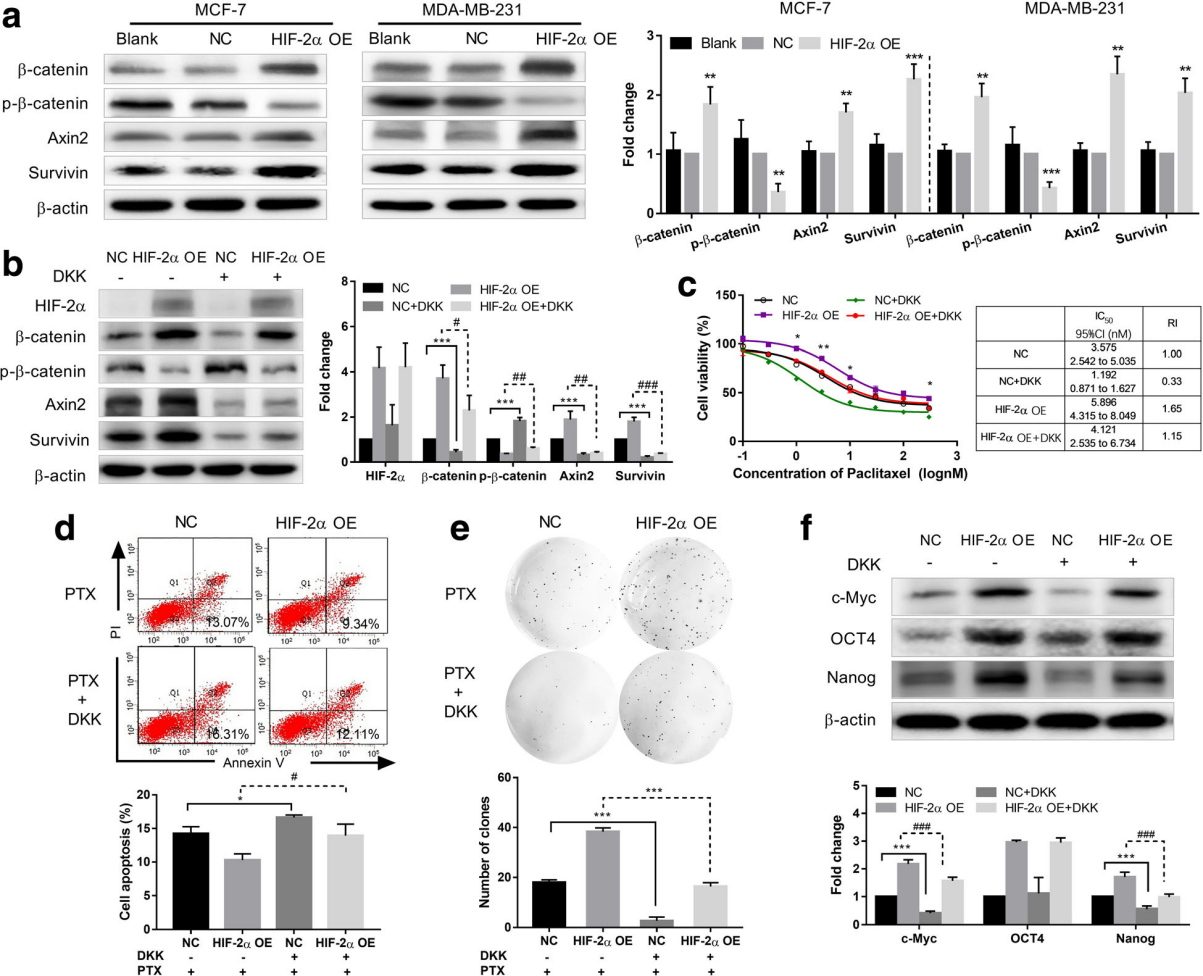
HIF-2α regulates stem phenotype conversion and drug resistance of breast cancer cells by activating the Wnt pathway. **a** Left: Expression of Wnt pathway-related proteins in HIF-2α–overexpressing MCF7 and MDA-MB-231 cells was detected by western blot. Right: Densitometric analysis of protein expression. **b** Left: Expression of HIF-2α and Wnt pathway-related proteins was detected after HIF-2α–overexpressing MCF7 cells were treated with DKK (100 ng/ml) for 48 h by western blot. Right: Densitometric analysis of protein expression. **c** Left: Cell viability was determined in HIF-2α–overexpressing MCF7 cells incubated with PTX combined with DKK (100 ng/ml) for 48 h using the MTT assay. Right: Comparison of IC_50_ values and resistance index. **d** Cell apoptosis was measured after HIF-2α–overexpressing MCF7 cells were incubated with PTX (3 nM) combined with DKK (100 ng/ml) for 48 h by flow cytometry. Quantification values are shown. **e** Self-renewal ability was analyzed after stable HIF-2α cDNA transfected MCF7 cells were incubated with PTX (3 nM) and DKK (100 ng/ml) for 48 h. **f** Expression of c-Myc, OCT4 and Nanog proteins was detected after HIF-2α–overexpressing MCF7 cells were treated with DKK (100 ng/ml) for 48 h by western blot. Quantification values are shown. Data are the mean ± SD of three independent experiments performed in triplicate. ^*^P < 0.05, ^**^P < 0.01, ^***^P < 0.0001 compared with the NC-cDNA group. ^#^*P* < 0.05, ^##^*P* < 0.01, ^###^*P* < 0.0001 compared with the HIF-2α-cDNA group

Given that c-Myc is an important transcription factor in regulating the stem phenotype and is also a common target gene of Wnt and Notch pathways, we inferred that the increased stem phenotype and resistance induced by HIF-2α was related to activation of Wnt and Notch pathways. To verify this hypothesis, we first measured changes in Wnt pathway-related proteins expressions in HIF-2α overexpressing MCF7 and MDA-MB-231 cells by western blot. Proteins expressions of β-catenin, survivin and axin2 were significantly increased, and p-β-catenin expression was decreased in HIF-2α overexpressing MCF7 and MDA-MB-231 cells compared to the NC group (Fig. 3a), suggesting that HIF-2α overexpression could activate the Wnt pathway in breast cancer cells. We also detected Wnt pathway proteins expressions with or without PTX treatment (3 nM and 100 nM for MCF7 and MDA-MB-231 cells, respectively) 48 h. As shown in Additional file 1: Figure S1, Wnt pathway has no obvious difference between with PTX and without PTX treatment. Besides, in HIF-2α silencing MCF7 MS cells, we detected Wnt pathway by western blot. We observed Wnt pathway related proteins decreased after HIF-2α silencing in MCF7 MS cells (Additional file 2: Figure S2d).

To further study whether HIF-2α induces conversion to a stem cell phenotype and resistance of breast cancer cells to PTX by activating the Wnt pathway and increasing c-Myc expression, we first treated HIF-2α overexpressing MCF7 cells with DKK (100 ng/ml), a Wnt pathway inhibitor, and detected changes in Wnt pathway-related proteins by western blot. Compared to the HIF-2α-cDNA group, β-catenin, survivin and axin2 expression was decreased, while p-β-catenin expression was increased in the HIF-2α-cDNA + DKK group. However, HIF-2α expression did not change (Fig. 3b). These results indicate that DKK reversed activation of the Wnt pathway induced by HIF-2α but did not impact HIF-2α expression.

We evaluated cell viability after HIF-2α-overexpressing MCF7 cells were treated with PTX combined with DKK (100 ng/ml) for 48 h. The cell viability rate in the HIF-2α-cDNA + DKK group was reduced compared to the HIF-2α-cDNA group (Fig. 3c). In addition, the early apoptosis rate in the HIF-2α-cDNA + DKK group was higher than in the HIF-2α-cDNA group based on flow cytometry analysis (Fig. 3d). These results strongly indicate that inhibiting the Wnt pathway could reverse the resistance of breast cancer cells to PTX induced by HIF-2α overexpression.

We detected changes of self-renew ability in HIF-2α-overexpressing MCF7 cells treated with PTX combined with DKK for 48 h. We found that the number and size of colonies were obviously decreased in HIF-2α-cDNA + DKK group compared with the HIF-2α-cDNA group (Fig. 3e). We also detected changes in expression of c-Myc, OCT4 and Nanog in HIF-2α-overexpressing MCF7 cells treated with DKK. We found that expression of c-Myc and Nanog was decreased, although there were no obvious changes in OCT4 expression in the HIF-2α-cDNA + DKK group compared with the HIF-2α-cDNA group (Fig. 3f). We therefore conclude that HIF-2α induces conversion of a stem cell phenotype in breast cancer cells and increases resistance to PTX by activating the Wnt pathway.

### HIF-2α overexpression increases stem-like properties and breast cancer cell resistance by activating the notch pathway

**Fig. 4:**
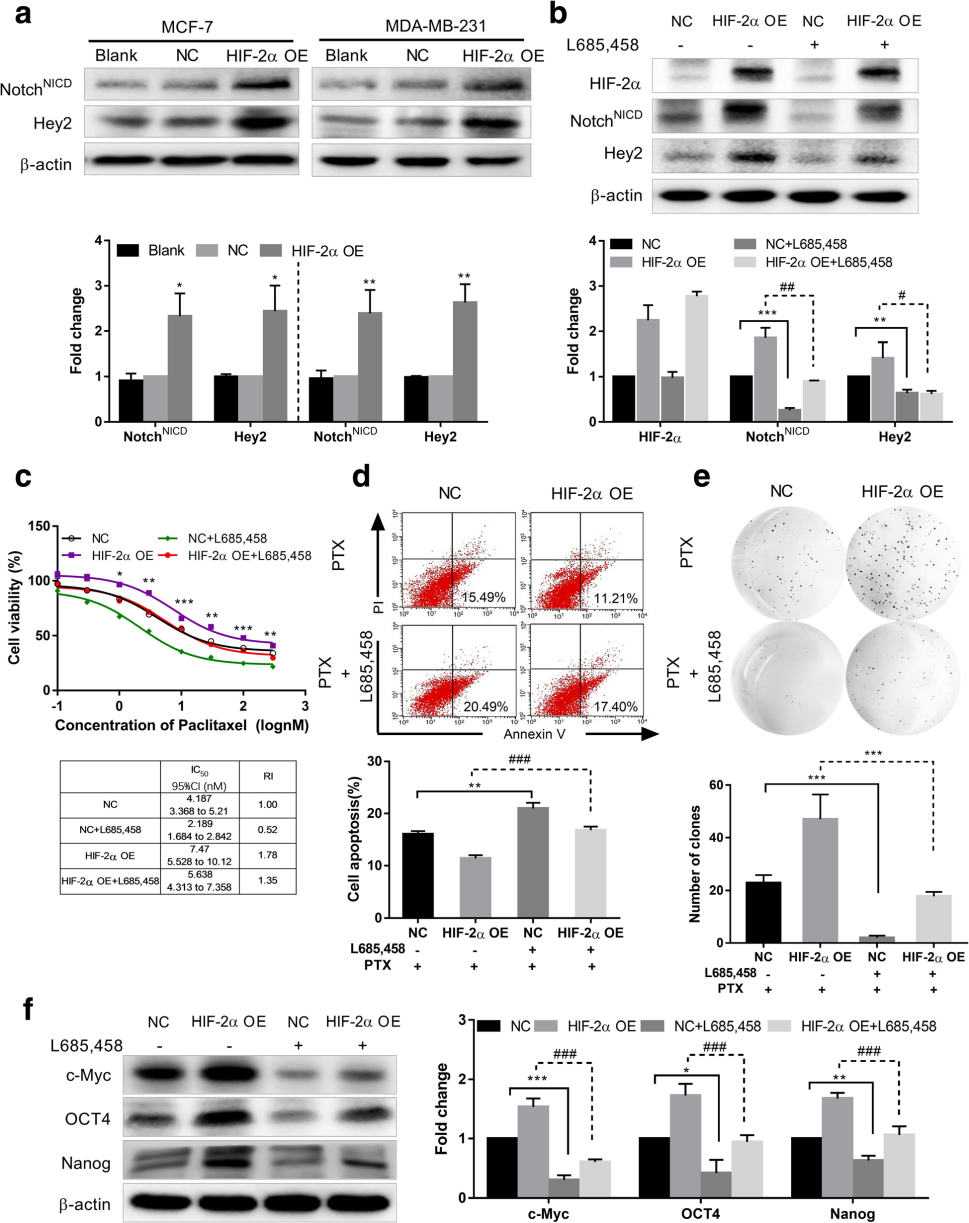
Overexpression of HIF-2α induces stem phenotype conversion and drug resistance by activating the Notch pathway. **a** Expression of Notch pathway-related proteins was detected in HIF-2α overexpressing MCF7 and MDA-MB-231 cells by western blot. **b** Expression of HIF-2α and Notch pathway-related proteins was detected after HIF-2α–overexpressing MCF7 cells were treated with L685,458 (2 μM) for 48 h by western blot. **c** Top: Cell viability was determined in HIF-2α–overexpressing MCF7 cells incubated with PTX and L685,458 (2 μM) for 48 h using the MTT assay. Bottom: Comparison of IC_50_ values and resistance index. **d** Cell apoptosis was measured after HIF-2α overexpressing MCF7 cells were incubated with PTX (3 nM) and L685,458 (2 μM) for 48 h by flow cytometry. Quantification values are shown. **e** Self-renew ability was analyzed after stable HIF-2α–cDNA transfected MCF7 cells were incubated with PTX (3 nM) and L685,458 (2 μM) for 48 h. **f** Expression of c-Myc, OCT4 and Nanog proteins was detected after HIF-2α–overexpressing MCF7 cells were treated with L685,458 (2 μM) for 48 h. Data are the mean ± SD of three independent experiments performed in triplicate. ^*^P < 0.05, ^**^P < 0.01, ^***^P < 0.0001 compared with the NC-cDNA group. ^#^*P* < 0.05, ^###^*P* < 0.0001 compared with the HIF-2α-cDNA group

To elucidate whether the enhanced stem phenotype and resistance to PTX in breast cancer cells induced by HIF-2α is dependent on Notch pathway activation, we also tested changes in Notch pathway-related proteins in HIF-2α–overexpressing MCF7 and MDA-MB-231 cells by western blot. Similar to previous results, expression of Notch^NICD^, an active factor in the Notch pathway, and Hey2, a downstream target gene, were both increased in HIF-2α–overexpressing MCF7 and MDA-MB-231 cells compared to the NC group (Fig. 4a). We also detected Notch pathway proteins expression with or without PTX treatment (3 nM and 100 nM for MCF7 and MDA-MB-231 cells, respectively) for 48 h. As shown in Additional file 1: Figure S1, Notch pathway has no obvious difference between with PTX and without PTX treatment. Additionally, in HIF-2α silencing MCF7 MS cells, we detected Notch pathway by western blot. We observed Notch pathway related proteins decreased after HIF-2α silence in MCF7 MS cells (Additional file 2: Figure S2e).

To further confirm this postulation, we treated HIF-2α-overexpressing MCF7 cells with L685,458 (2 μM), a Notch pathway inhibitor, and determined changes in Notch pathway-related proteins. We found that Notch^NICD^ and Hey2 expression was decreased in the HIF-2α-cDNA + L685,458 group compared to the HIF-2α-cDNA group. HIF-2α expression did not notably change (Fig. 4b). These data suggest that L685,458 reversed activation of the Notch pathway induced by HIF-2α overexpression but did not impact HIF-2α expression.

We also evaluated cell viability after HIF-2α-overexpressing MCF7 cells were treated with PTX combined with L685,458 (2 μM) for 48 h. The cell viability rate in the HIF-2α-cDNA + L685,458 group was decreased compared to the HIF-2α-cDNA group (Fig. 4c). In addition, the early apoptosis rate in the HIF-2α-cDNA + L685,458 group was increased relative to the HIF-2α-cDNA group by flow cytometry analysis (Fig. 4d). These findings suggest that inhibiting the Notch pathway could reverse the resistance of breast cancer cells to PTX induced by HIF-2α overexpression.

We detected changes of self-renew ability in HIF-2α-overexpressing MCF7 cells treated with PTX combined with L685,458 for 48 h. We found that the number and size of colonies were obviously decreased in HIF-2α-cDNA + L685,458 group compared with the HIF-2α-cDNA group (Fig. 4e). We further measured the expression of c-Myc, OCT4 and Nanog in HIF-2α-overexpressing MCF7 cells treated with L685,458. Compared with the HIF-2α-cDNA group, the expression of c-Myc, OCT4 and Nanog were all decreased in the HIF-2α-cDNA + L685,458 group (Fig. 4f). Thus, we conclude that HIF-2α induces conversion to a stem cell phenotype in breast cancer cells and increases resistance to PTX by activating the Notch pathway.

### HIF-2α activates the Wnt and notch pathways to increase tumorigenicity and resistance of breast cancer cells to PTX in vivo

**Fig. 5:**
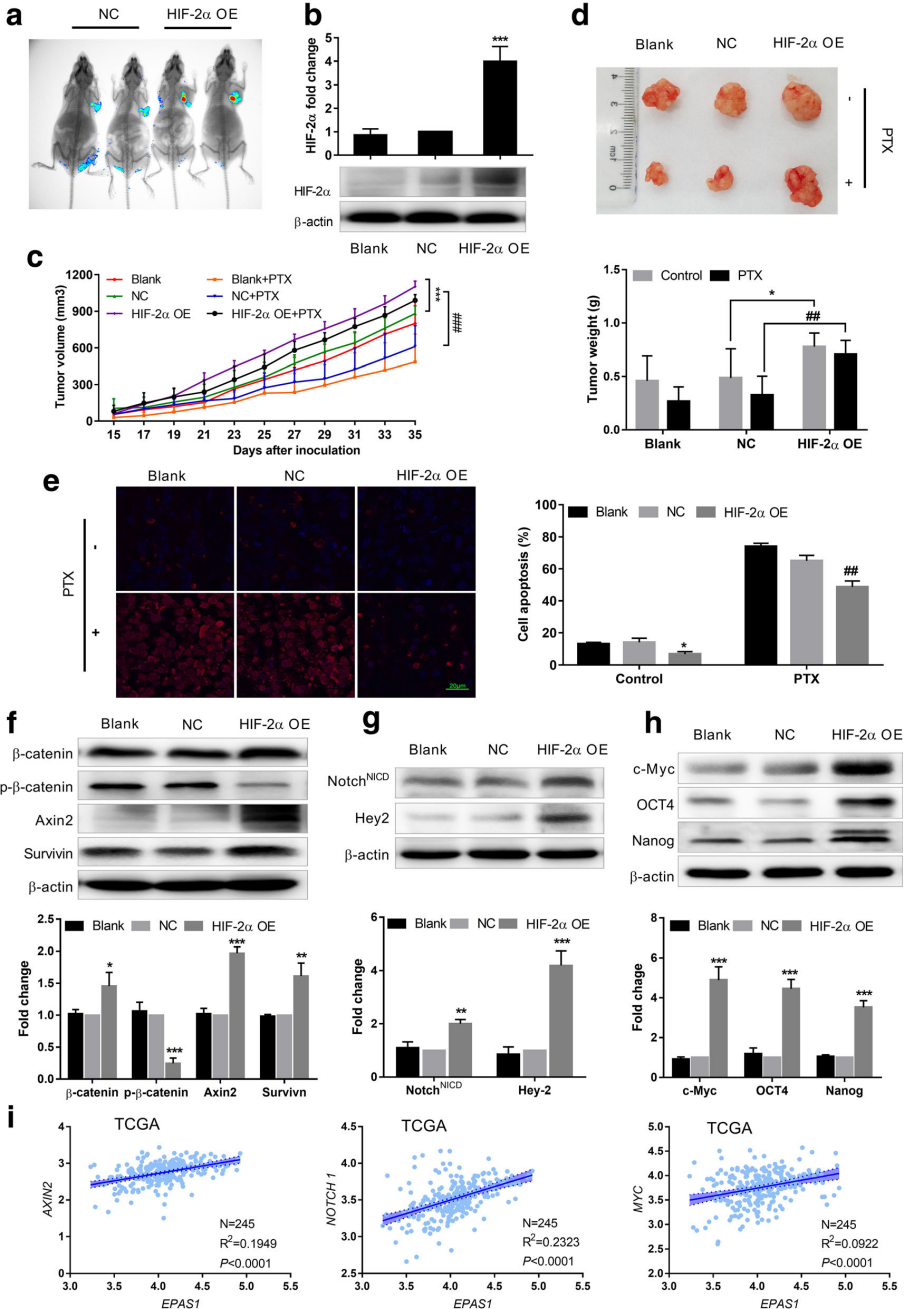
HIF-2α overexpression increases tumorigenicity and resistance to PTX by activating the Wnt and Notch pathways in vivo. **a** Green fluorescent protein (GFP) expression was detected in xenograft mice stably transfected with NC-cDNA and HIF-2α-cDNA MCF7 cells by small animal imaging. **b** HIF-2α expression was measured by western blot in xenograft tumor tissues. **c** Average tumor volumes were measured in xenograft mice every two days. **d** Images of resected MCF7 tumor tissues and average tumor weight at the end of indicated treatment. **e** TUNEL measurement of apoptosis in xenograft mice using fluorescent staining after treatment with or without PTX. Red fluorescence  cells were considered positive (magnification × 600). Bar standards are 20 μm. **f** Expression of Wnt pathway-related proteins was detected by western blot in different treatment groups. **g** Expression of Notch pathway-related proteins was detected by western blot in different treatment groups. **h** Expression of c-Myc, OCT4 and Nanog was determined by western blot in different treatment groups. **i** Correlation analysis of the *EPAS1* and Wnt, Notch pathway key factors in TCGA dataset. *AXIN2*, *NOTCH1* and *MYC* display significant positive correlation with *EPAS1* expression (*p* < 0.0001) in TCGA datasets. Data are shown as the mean ± SD (*n* = 6), ^*^P < 0.05, ^**^P < 0.01, ^***^P < 0.0001 compared with the NC-cDNA group. ^##^*P* < 0.01 compared with the NC-cDNA + PTX group

To verify the effect of HIF-2α overexpression in inducing a stem phenotype and resistance to PTX by activating the Wnt and Notch pathways in vivo, we constructed a HIF-2α–overexpressing MCF7 cell and HIF-2α–overexpressing MDA-MB-231 cell mouse xenograft models. As shown in Fig. 5a and Additional file 3: Figure S3, xenograft tumors from stably transfected NC-cDNA and HIF-2α-cDNA MCF7 and MDA-MB-231 cells all expressed green fluorescent protein (GFP). We also detected increased HIF-2α expression in tumor tissues in the HIF-2α-cDNA group using western blot analysis (Fig. 5b). All of the results revealed that the xenograft model was successfully constructed.

We then evaluated changes in tumorigenicity and resistance to PTX induced by HIF-2α in vivo. Xenograft mice in the HIF-2α-cDNA group had larger tumor volumes and weights compared with the NC-cDNA group (Fig. 5c, d, Additional file 3: Figure S3b, c), suggesting that HIF-2α increased the tumorigenicity of breast cancer cells. In addition, tumor volumes and weights in the HIF-2α-cDNA + PTX (5 mg/kg, every two days, i.p.) mice were notably increased than those in NC-cDNA + PTX mice (Fig. 5c, d, Additional file 3: Figure S3b, c), suggesting that HIF-2α increased the resistance of breast cancer cells to PTX in vivo.

To evaluate whether HIF-2α overexpression impacts apoptosis in vivo, we detected cell apoptosis in tumor tissues using a TUNEL assay. The percentage of apoptotic cells was increased in NC-cDNA + PTX xenograft tumors compared to NC-cDNA xenograft tumors. The percentage of apoptotic cells was decreased in HIF-2α-cDNA + PTX xenograft tumors compared to NC-cDNA + PTX xenograft tumors (Fig. 5e), indicating that HIF-2α overexpression can decrease apoptosis induced by PTX, which means that breast cancer cells are more resistant to PTX in vivo.

We detected changes in Wnt and Notch pathway-related proteins in xenograft tumors by western blot. Activation of the Wnt pathway was associated with increased expression of β-catenin, axin2 and survivin and decreased p-β-catenin expression (Fig. 5f), while activation of the Notch pathway was linked to increased expression of Notch^NICD^ and Hey2 in HIF-2α-cDNA xenograft tumors (Fig. 5g). As expected, expression of c-Myc, OCT4 and Nanog was higher in the HIF-2α-cDNA group than the NC-cDNA group (Fig. 5h). Similar to our previous in vitro results, we demonstrated that HIF-2α activated Wnt and Notch pathways to increase tumorigenicity and resistance of breast cancer cells to PTX in vivo.

Finally, we selected 245 cases CD44^+^CD24^−^ (breast cancer stem cells phenotype marker) breast cancer patients from TCGA dataset to analyze correlation of *EPAS1* with Wnt and Notch pathways related factors, and c-Myc. As shown in Fig. 5i, *EPAS1* exhibited positive correlation with *AXIN2*, *NOTCH1* and *MYC*. This result further proved HIF-2α, c-Myc, Wnt and Notch pathway ways were all activated in BCSCs.

## Discussion

Tumor hypoxia is associated with high grade malignancy, poor prognosis and resistance to radiotherapy and chemotherapy. Under various hypoxic microenvironments, the transcription factors HIF-1α and HIF-2α regulate different transcriptional responses to hypoxic stress [16]. In our study, HIF-1α protein expression was obviously increased and HIF-2α expression was slightly increased in MCF7 cells under 1% O_2_ for 6–12 h. In contrast, HIF-1α expression began to decline while HIF-2α gradually accumulated from 24 to 48 h. Holmquist et al. also reported that increased HIF-1α protein levels had degraded after 8 h of hypoxia (1% O_2_), while HIF-2α protein levels continuously accumulated with time in neuroblastoma cells [26]. Similarly, Uchida et al. reported that the induced HIF-1α protein disappeared, whereas HIF-2α protein levels remained high and stable with prolonged hypoxia (0.5% O_2_, 12 h) in lung cancer A549 cells [27]. Although these periods of chronic hypoxia are different, we found that HIF-2α accumulated under chronic hypoxia, which is consistent with these reports. In this study, we detected changes in the resistance of breast cancer cells to PTX under hypoxic conditions for different periods of time. Prolonged hypoxia (48 h) increased the resistance of MCF7 cells to PTX along with increased HIF-2α expression, which suggests that HIF-2α overexpression induced by chronic hypoxia may be a crucial factor in mediating breast cancer cell resistance to PTX.

BCSCs are regarded as source of tumor maintenance and recurrence [28]. Recently,more and more studies have validated that cancer stem cells are critically depended on hypoxia microenvironment and HIFs play an important role in patient poor survival and therapeutic resistance [29]. As we all known, cancer stem cells are a very small subpopulation in tumor cells. For the left major tumor cells, how hypoxia environment regulated them performing chemoresistance? In our opinion, cancer stem cell phenotype conversion is an important process.

Cancer stem cell phenotype conversion, also called phenotype plasticity, generally describes dynamic processes in which cancer cells are endowed with stem cell-like traits, such as self-renew ability, EMT and chemoresistance [30, 31]. Hypoxia is involved in inducing phenotypic plasticity in cancer stem cells and therapy resistance [30]. However, whether HIF-2α participates in this transforming process and modulates chemotherapy resistance has not been reported. MCF7 and MDA-MB-231 cells were different phenotype breast cancer cells. MCF7 cells are ER^+^, PR^+^, Her2^−^ Lumina A phenotype breast cancer cells and MDA-MB-231 cells are ER^−^, PR^−^, Her2^−^ basal-like breast cancer cells. We aimed to target HIF-2α to study the mechanisms of hypoxia environment mediating chemoresistance in different phenotype breast cancer cells to expand clinical application scope in the future. In the present study, we found that HIF-2α overexpression could increase MCF7 and MDA-MB-231 cell resistance to PTX, anti-apoptosis and expression of the cancer stem cell markers OCT4 and Nanog. These findings demonstrate that HIF-2α can enhance the stemness phenotype of breast cancer cells to induce resistance to PTX. Alam et al. reported that HIF-2α contributed to antiestrogen resistance via positive bilateral crosstalk with EGFR in breast cancer cells [32]. However, mechanisms in the resistance of breast cancer cells to PTX induced by HIF-2α remain unclear.

The Wnt pathway participated in cancer stem cell phenotype conversion. Hypoxia can activate Wnt5A to induce melanoma stem phenotypic plasticity [31]. It was reported that HIF-2α can induce Wnt10b expression to regulate pluripotency, cell proliferation and differentiation in adipogenic cells [33]. Choi et al. demonstrated that HIF-2α interacted with β-catenin and facilitated gene transcription to promote proliferation of clear cell renal cell carcinoma cells [34]. However, in breast cancer cells, whether HIF-2α can mediate stem phenotype conversion to induce resistance to PTX via the Wnt pathway has not been reported. In the present study, we found that HIF-2α could activate the Wnt pathway in MCF7 and MDA-MB-231 cells. DKK, a Wnt pathway inhibitor, reversed the resistance of breast cancer cells to PTX, anti-apoptosis, and high expression of stem cell markers induced by HIF-2α overexpression, which confirms that HIF-2α induced stem cell phenotype conversion and resistance to PTX by activating the Wnt pathway.

The Notch signaling pathway is another fundamental pathway that maintains the stemness of BCSCs [35, 36]. We found that HIF-2α induced high expression of Notch^NICD^ and Hey2 in MCF7 and MDA-MB-231 cells. L685,458, an inhibitor of the Notch pathway, inhibited activation of the Notch pathway and reversed resistance to PTX, anti-apoptosis and high expression of cancer stem cell markers induced by HIF-2α overexpression. These data demonstrate that HIF-2α can activate the Notch pathway to induce a stem phenotype and increase resistance of breast cancer cells to PTX. Hu et al. reported that HIF-2α directly interacts with Notch^NICD^ and represses activity of the Notch pathway in glioma stem cells [37]. However, many functions of the various signaling pathways are different in glioma stem cells than in other cancer cell types. This may be one of the reasons HIF-2α regulates the Notch pathway in different ways.

Besides, we also detected shh pathway and found shh pathway was not activated after HIF-2α overexpression (data not shown). Past, we have reported shh pathway was activated in BCSCs and play a more important role than Wnt pathway in MCF7 MS cells [38]. Based our past study and recent data, we inferred that shh pathway was innate activation in breast cancer stem cells (MCF7 MS cells) and maybe didn’t function in parental breast cancer cells (MCF7 cells), but Wnt and Notch pathways should play a more important role in stem phenotype switch process. Although three of them were all critical pathways in stem cells and cancer stem cells, their specific mechanisms were rather different.

Oncogenic c-Myc plays an important role in inducing a stem cell phenotype [39]. Our study found that HIF-2α overexpression up-regulated c-Myc expression in breast cancer cells. In different cancers, HIF-2α has shown different modulations on c-Myc expression. Gordan et al. reported that HIF-2α promoted c-Myc transcriptional activity and mediated tumorigenesis in renal clear cell carcinoma [11]. In human pulmonary endothelial cells, HIF-2α attenuated c-Jun activation and suppressed c-Myc expression [40]. Indeed, recent reports have verified there was cross-talk between the Wnt and Notch pathways. In different cell lines, the interaction of Wnt and Notch pathways were rather different. Sometimes, they activate each other, but sometimes they suppress each other [41, 42]. In our study, we have verified that HIF-2α could activate Wnt and Notch pathways. The two pathways both activated downstream target gene c-Myc to promoted breast cancer stemness phenotype conversion. But under hypoxia condition, dose HIF-2α regulate Wnt and Notch pathways respectively or by cross talk between Wnt and Notch pathways? This problem needs to be further studied.

Briefly, the present study demonstrates that HIF-2α can activate Wnt and Notch pathways to up-regulate c-Myc expression, which enhances the stem phenotype and induces breast cancer cells chemoresistance to PTX.

## Conclusions

The present study found that chronic hypoxia enhanced breast cancer cell resistance to PTX and induced high expression of HIF-2α. Further investigation demonstrated that HIF-2α induced stem phenotype conversion and promoted the resistance of breast cancer cells to PTX by activating the Wnt and Notch, but not Shh included, pathways. These findings offer a new mechanism of resistance and a novel target for breast cancer therapy in clinical studies.

## Additional files

Additional file 1: **Figure S1.** PTX had no effect on HIF-2α up-regulating cancer stem cell markers, Wnt and Notch pathways. Expression of c-Myc, OCT4, Nanog, Wnt and Notch pathways in HIF-2α silencing MCF7 MS cells was detected with or without PTX treatment by western blot. (JPG 483 kb)

Additional file 2: **Figure S2.** HIF-2α silence decreases the stemness phenotype and activation of Wnt and Notch pathways of breast cancer stem cells, **a** Left: Cell viability of stable HIF-2α silencing MCF7 MS cells after treatment with PTX (0–300 nM) for 48 h was measured using the MTT assay. Right: Comparison of IC_50_ values. **b** The self-renew ability of stable HIF-2α silencing MCF7 MS cells with or without PTX (3 nM) treatment for 48 h was measured by mammosphere formation assay. **c** Expression of c-Myc, OCT4 and Nanog proteins in the HIF-2α-silenced MCF7 MS cells was detected by western blot. **d** Expression of Wnt pathway-related proteins in the HIF-2α-silenced MCF7 MS cells was detected by western blot. **e** Expression of Notch pathway-related proteins in the HIF-2α-silenced MCF7 MS cells was detected by western blot. (JPG 862 kb)

Additional file 3: **Figure S3.** HIF-2α overexpression increases tumorigenicity and resistance to PTX. **a** Green fluorescent protein (GFP) expression was detected in xenograft mice stably transfected with NC-cDNA and HIF-2α-cDNA MDA-MB-231 cells by small animal imaging. **b** Average tumor volumes were measured in xenograft mice every two days. **c** Images of resected MDA-MB-231 tumor tissues and average tumor weight at the end of indicated treatment. (JPG 522 kb)

## Abbreviations

ARNT: Aryl hydrocarbon receptor nuclear translocator
HIFs: Hypoxia inducible factor
PTX: Paclitaxel
RI: Resistance index
TME: Tumor microenvironment
