# Supplementary figures and images for: HIF-2α promotes conversion to a stem cell phenotype and induces chemoresistance in breast cancer cells by activating Wnt and Notch pathways
Source: J Exp Clin Cancer Res. 2018 Oct 19;37:256. doi: 10.1186/s13046-018-0925-x (PMC6194720; doi:10.1186/s13046-018-0925-x)

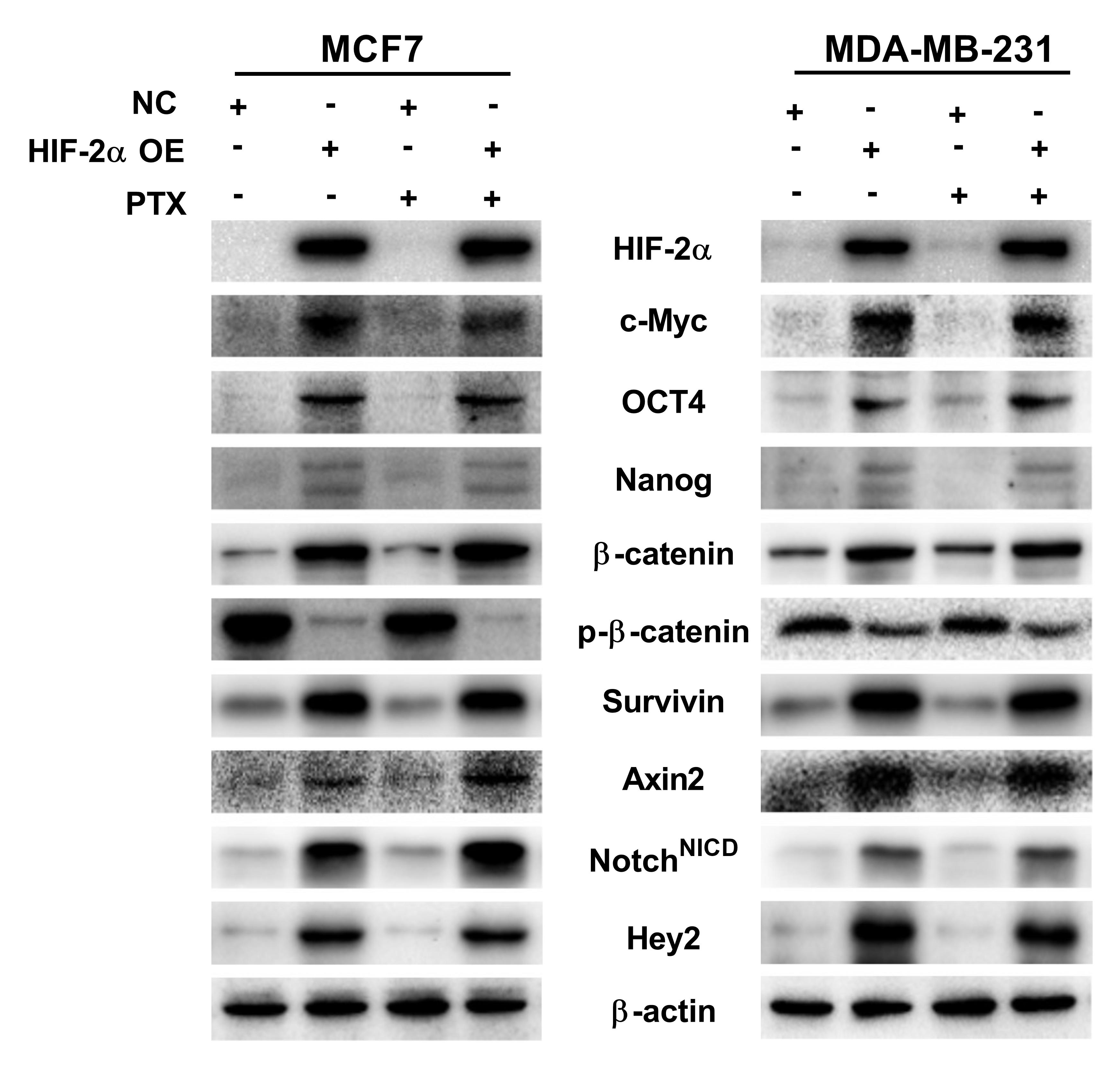

Supplement: Supplementary file 1 — Figure S1. PTX had no effect on HIF-2α up-regulating cancer stem cell markers, Wnt and Notch pathways. Expression of c-Myc, OCT4, Nanog, Wnt and Notch pathways in HIF-2α silencing MCF7 MS cells was detected with or without PTX treatment by western blot. (JPG 483 kb) [file 13046_2018_925_MOESM1_ESM.jpg]

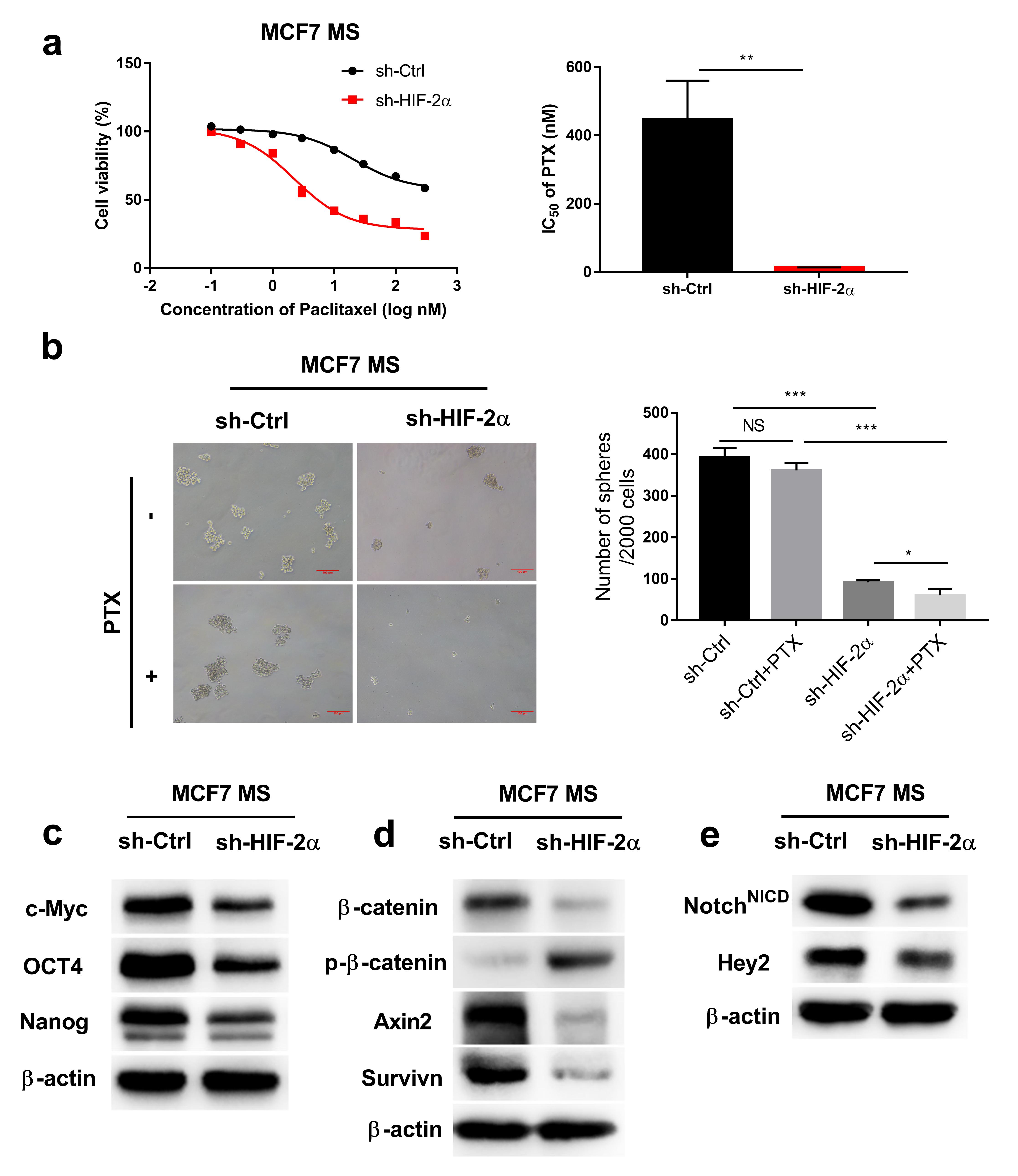

Supplement: Supplementary file 2 — Figure S2. HIF-2α silence decreases the stemness phenotype and activation of Wnt and Notch pathways of breast cancer stem cells, a Left: Cell viability of stable HIF-2α silencing MCF7 MS cells after treatment with PTX (0–300 nM) for 48 h was measured using the MTT assay. Right: Comparison of IC50 values. b The self-renew ability of stable HIF-2α silencing MCF7 MS cells with or without PTX (3 nM) treatment for 48 h was measured by mammosphere formation assay. c Expression of c-Myc, OCT4 and Nanog proteins in the HIF-2α-silenced MCF7 MS cells was detected by western blot. d Expression of Wnt pathway-related proteins in the HIF-2α-silenced MCF7 MS cells was detected by western blot. e Expression of Notch pathway-related proteins in the HIF-2α-silenced MCF7 MS cells was detected by western blot. (JPG 862 kb) [file 13046_2018_925_MOESM2_ESM.jpg]

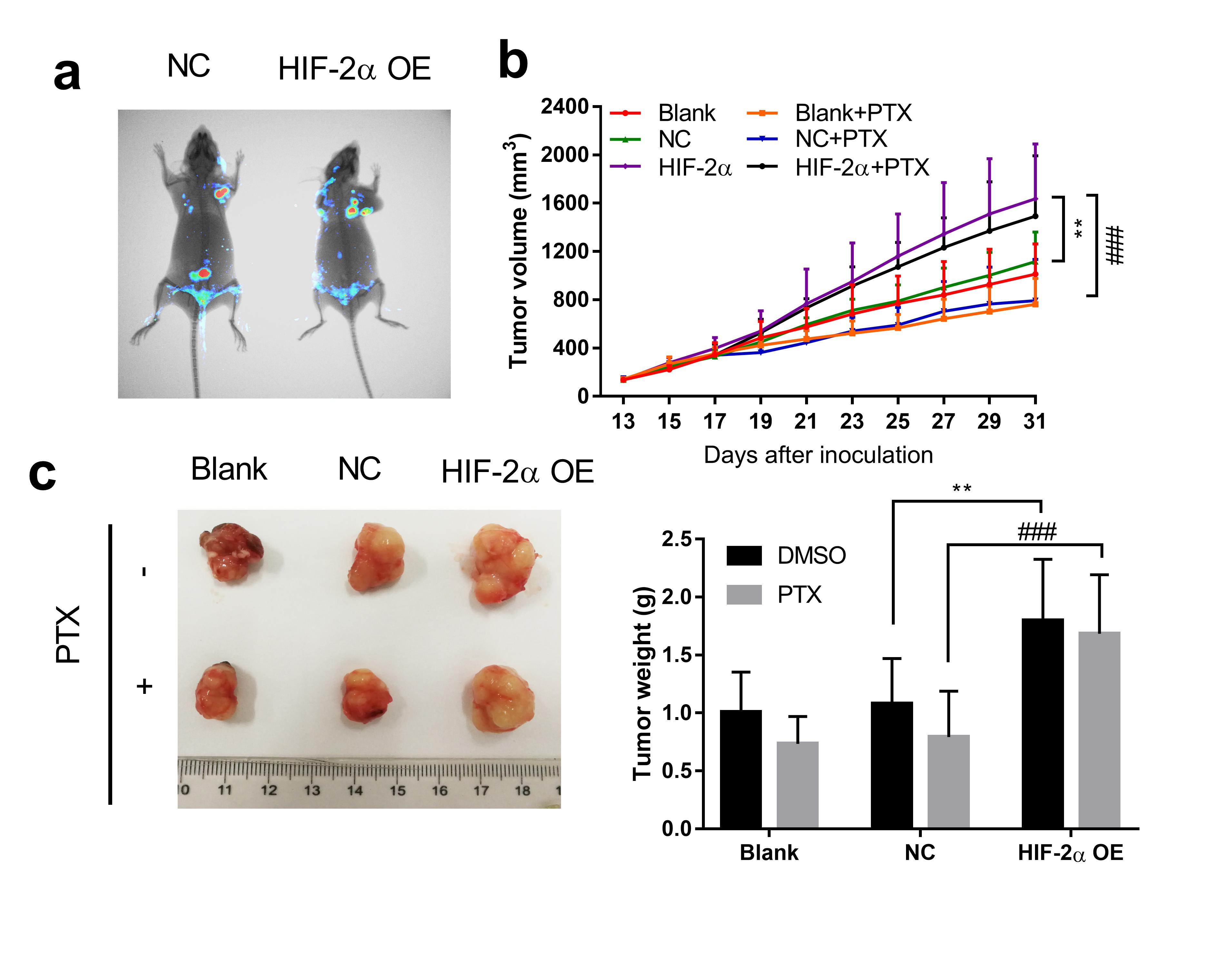

Supplement: Supplementary file 3 — Figure S3. HIF-2α overexpression increases tumorigenicity and resistance to PTX. a Green fluorescent protein (GFP) expression was detected in xenograft mice stably transfected with NC-cDNA and HIF-2α-cDNA MDA-MB-231 cells by small animal imaging. b Average tumor volumes were measured in xenograft mice every two days. c Images of resected MDA-MB-231 tumor tissues and average tumor weight at the end of indicated treatment. (JPG 522 kb) [file 13046_2018_925_MOESM3_ESM.jpg]
